# Pleuropulmonary Blastoma: A Case Report and Review of the Literature

**DOI:** 10.1155/2014/509086

**Published:** 2014-08-07

**Authors:** Amjad Ali Khan, Ahmed Kamal El-Borai, Mohammad Alnoaiji

**Affiliations:** Prince Salman Armed Forces Hospital, Tabuk, Saudi Arabia

## Abstract

The case of 38-month-old boy is being reported who was brought to the pediatrics clinic with fever, cough, hemoptysis, and breathing difficulty. Imaging studies revealed a right lower chest mass. Lobectomy and histopathological examination revealed it to be predominantly solid pleuropulmonary blastoma type II. It is a rare pediatric pleuropulmonary tumor with aggressive behavior and tendency to spread to the brain. The case is being presented to make the general histopathologist aware of this rare entity and to highlight to the pediatric physicians/surgeons, radiologists, and histopathologists the fact that lung cysts in infants and young children should be evaluated seriously and sampled thoroughly to diagnose cases of type I pleuropulmonary blastoma which will progress over time to type II or type III tumors. Also the siblings and first degree relatives of the patient should be screened for associated pulmonary and extrapulmonary benign and malignant conditions.

## 1. Introduction

Pleuropulmonary blastoma (PPB) is a rare, aggressive, dysontogenetic, and malignant tumor of intrathoracic (pulmonary, pleural, or combined) mesenchyme [1, 2]. It was first reported in a study of 11 patients by Manivel et al. in 1988 [3]. PPB affects infants and children under four years of age [4]. Morphologically, PPB has three types (I, II, and III) and a fourth type (Ir) was added in 2006 [5].

This case is being reported with an aim to increase the general awareness for this entity so as to recognize this rare entity early in its course, since type I tumors (resembling congenital lung cyst) can progress over time to aggressive type II and type III tumors. Therefore, early histological recognition and differentiation from congenital airway malformations and other benign cysts are very important.

## 2. Case Presentation

**Figure 1:**
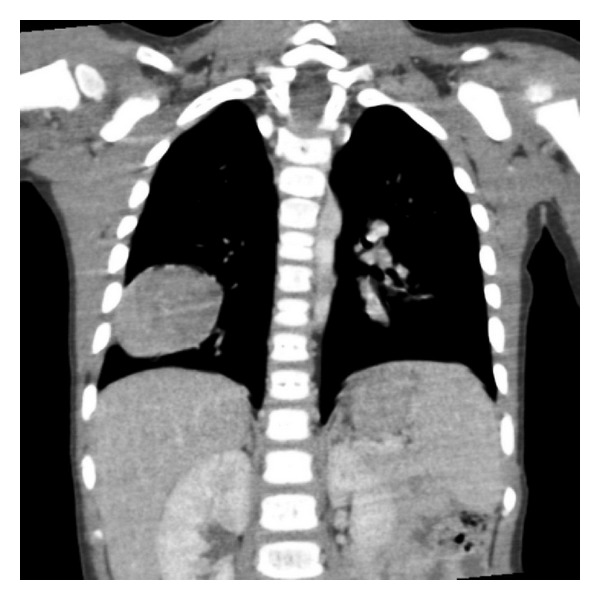
CT chest: right lung lower lobe peripheral mass.

**Figure 2:**
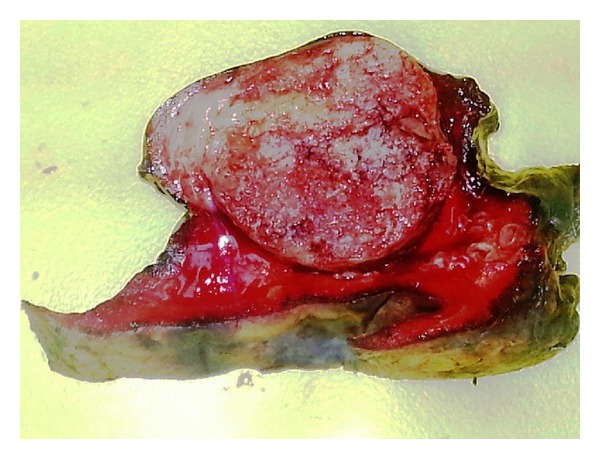
Circumscribed solid tumor mass surrounded by lung tissue.

**Figure 3:**
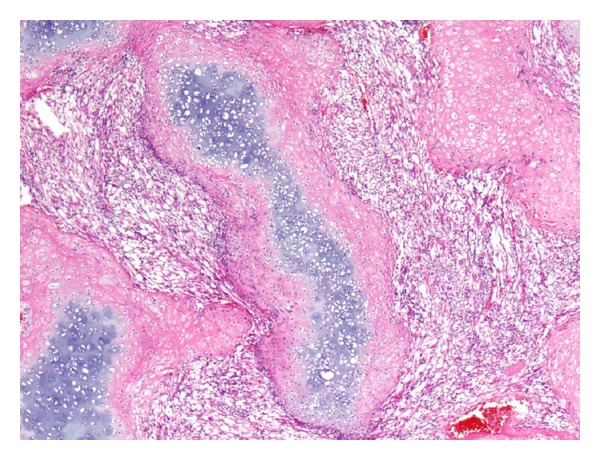
Islands of mature and immature hyaline cartilage. H&E: ×100.

**Figure 4:**
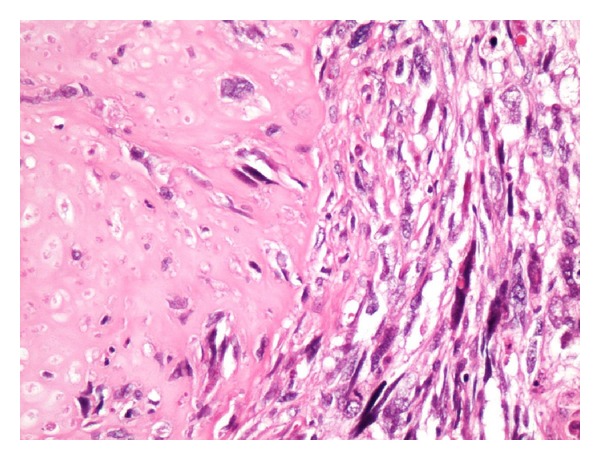
Interface of chondrosarcoma and rhabdomyosarcoma. H&E: ×200.

**Figure 5:**
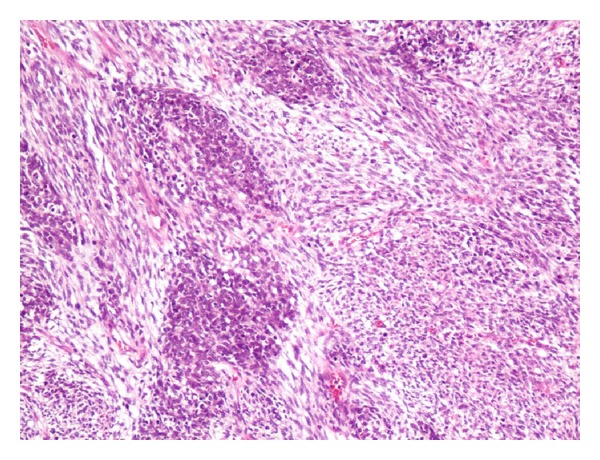
Nodules of blastema-like cells separated by loose tissue. H&E: ×100.

**Figure 6:**
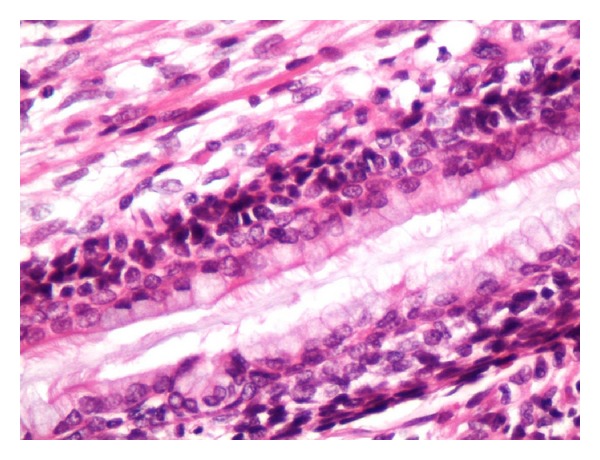
Ciliated columnar epithelium lining the microcyst and underlying condensation of small round cells. H&E: ×200.

**Figure 7:**
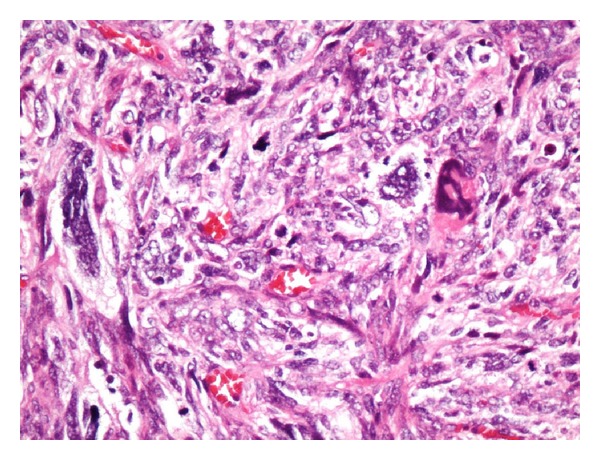
Anaplastic large bizarre cells amidst malignant pleomorphic cells. H&E: ×200.

**Figure 8:**
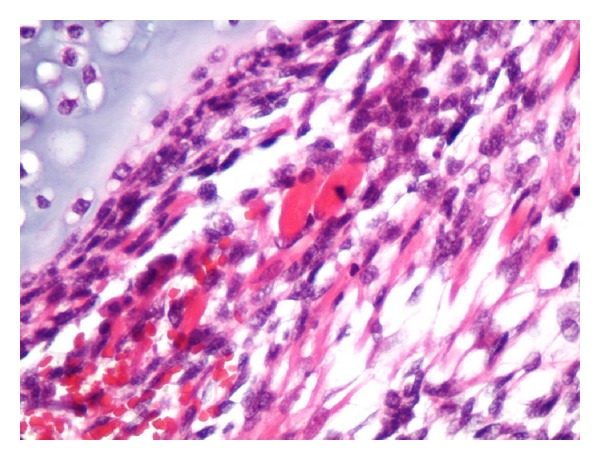
Scattered ovoid eosinophilic rhabdomyoblasts. H&E: ×200.

**Figure 9:**
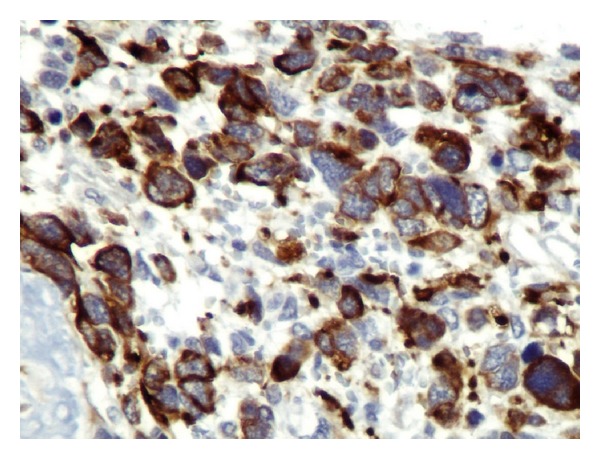
Desmin: cytoplasmic staining: ×400.

**Figure 10:**
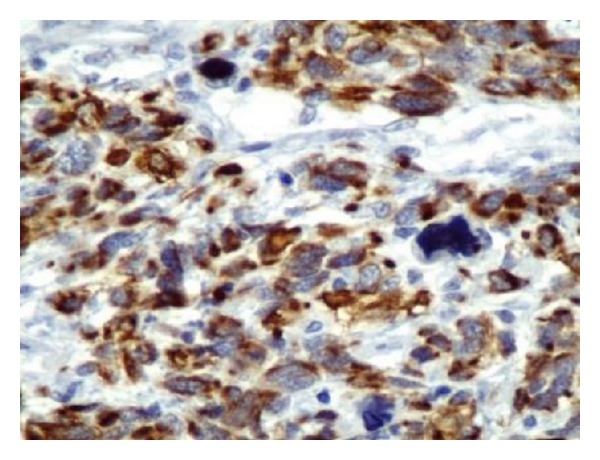
MSA: cytoplasmic staining: ×200.

**Figure 11:**
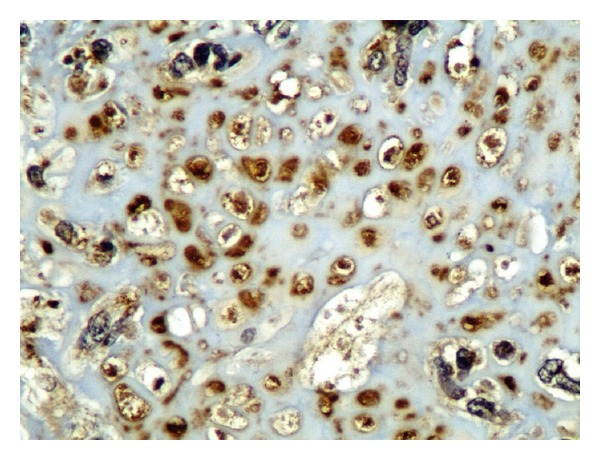
S100: nuclear staining in cartilage tissue: ×200.

**Figure 12:**
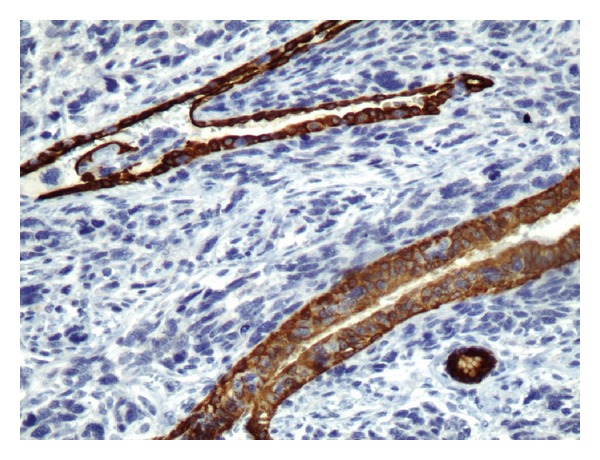
CK19 cytoplasmic staining of cyst lining epithelial cells: ×200.

**Figure 13:**
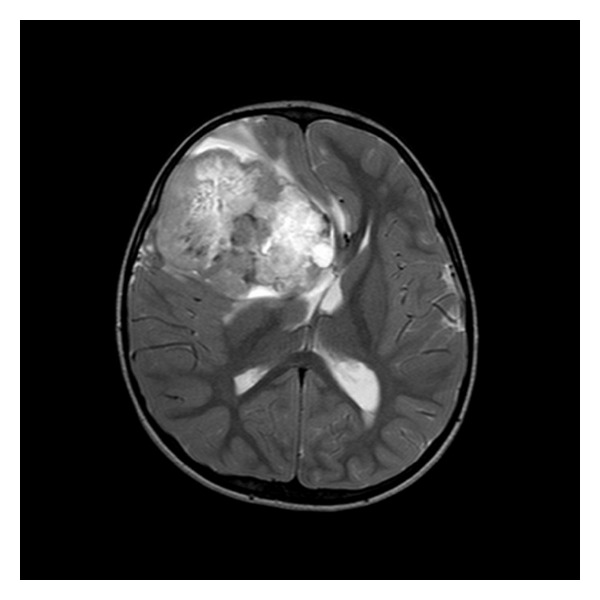
MRI brain: large right frontal lobe mass.

**Figure 14:**
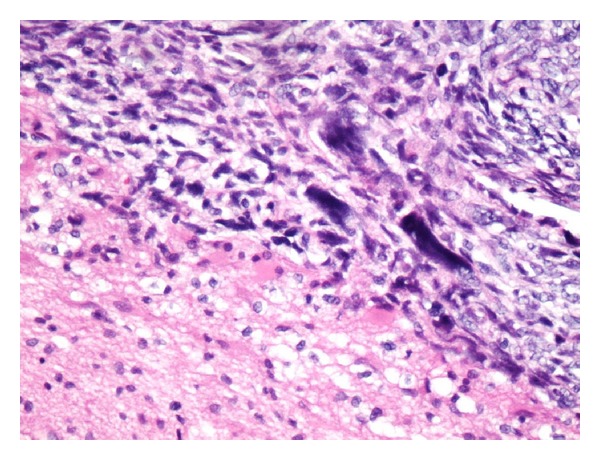
Metastatic sarcoma and edematous brain tissue interface. H&E: ×200.

**Figure 15:**
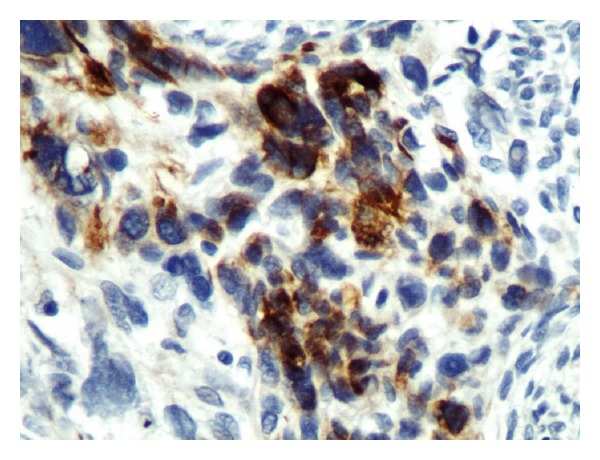
Brain metastasis, Desmin cytoplasmic staining: ×400.

A 38-month-old boy was brought to the pediatrics clinic with fever, cough, hemoptysis, and breathing difficulty. His chest radiograph and computerized tomography (CT) scan (Figure 1) showed a right lung lower lobe heterogeneous mass (3.9 × 3.3 × 3.2 cm) and right middle lobe atelectasis without pleural effusion. CT guided biopsy was performed and a single biopsy core was obtained. It was composed entirely of hyaline cartilage; the chondrocytes were increased in number and showed mild nuclear pleomorphism, no admixture of soft tissue, inflammation, or necrosis. Therefore, a differential diagnosis of chondroma, chondroid hamartoma, and pleuropulmonary blastoma was raised and lobectomy was suggested. Right thoracotomy was performed; the mass was found to be limited to the right lower lobe without involvement of the pleura. Right lower lobectomy was performed without complications. The excised mass was solid, circumscribed, firm, and gray-white measuring 4.2 × 3.2 × 3 cm surrounded by congested and partly solidified lung tissue. Its cut surface was heterogeneously gray-white with focal areas of hemorrhages and cartilaginous tissue (Figure 2). Microscopically, it was composed of many lobules of hyaline cartilage (Figure 3) in which the constituent chondrocytes varied from bland to moderately pleomorphic (Figure 4) with areas of necrosis (10%). The intervening stroma varied from blastema-like to fibromyxoid to dense spindle cell type (Figure 5), harboring an occasional microscopic cyst lined by ciliated cuboidal to columnar cells with subepithelial condensation of blastema-like to spindle shaped neoplastic cells (Figure 6). There were focal areas of skeletal muscle differentiation, bizarre multinucleate giant cells, and many scattered mitoses throughout (Figures 7 and 8). The surrounding pulmonary tissue showed areas of hemorrhages and atelectasis; the tumor was abutting but not involving the pleura. A small peribronchial lymph node did not show any tumor infiltration. Immunohistochemically, the rhabdomyoblasts were positive for Desmin and muscle specific actin (MSA) (Figures 9 and 10). The chondrocyte nuclei were positive for S100 (Figure 11) and the epithelium lining the cysts was positive for CK19 (Figure 12). In view of a predominantly solid pattern with scattered occasional microcysts, a diagnosis of pleuropulmonary blastoma type III was made and the material was sent to The International Pleuropulmonary Blastoma Registry, Minnesota, USA, for confirmation. There it was confirmed to be pleuropulmonary blastoma and retyped as predominantly solid type II tumor (due to the presence of microcysts). The parents of the child were informed and the treatment options were discussed; however, they refused any further treatment as the child had apparently improved symptomatically and wanted more time to decide. Three months later the patient was brought back to the oncology clinic with complaints of headache, vomiting, and dizziness. CT and magnetic resonance imaging (MRI) of the brain showed an intra-axial right frontal lobe mass measuring 7.3 × 4.2 × 4 cm (not apparent in the brain CT done three months earlier) with central area of necrosis causing compression of the ipsilateral lateral ventricle and significant midline shift (Figure 13). Craniotomy was performed and the resected mass was received in the laboratory as multiple fragments collectively measuring 7 × 5 × 1 cm. Microscopically, the tumor was composed of dense spindle cell proliferation with areas of hemorrhages, necrosis, and scattered anaplastic bizarre giant cells (Figure 14). The tumor cells were positive for Desmin and MSA (Figure 15). The patient left the hospital in a conscious and ambulatory state awaiting chemotherapy but was readmitted after a week with massive intracranial hemorrhage (right frontal lobe) and breathed his last after two days of hospitalization.

## 3. Discussion

Pleuropulmonary blastoma is a rare intrathoracic tumor accounting for 15% of all primary pediatric pulmonary tumors and 25% of these cases occur in familial settings [4]; the associated malignancies in first- and second-degree relatives include rhabdomyosarcomas, synovial sarcoma, pleuropulmonary blastoma, thyroid carcinomas, ovarian Sertoli-Leydig cell tumors, gonadal germ cell tumors, and certain types of leukemia [2, 4, 6, 7]. Associated benign conditions include thyroid nodule, cystic nephroma, colonic polyps, benign eye and nasal tumors, and neurofibromatosis [4–7]. The age of presentation is usually less than 4 years; very few cases have been reported in patients over 10 years of age and only one case has been reported at 36 years [4]. The median age of presentation depends largely on the tumor type. For type I tumors, the median age is 10 months; for type Ir, the median age is 48 months; for type II, the median age is 34 months; and for type III, the median age is 44 months [5]. There is no gender predilection and the tumor occurs more commonly on the right side [1]. Certain genetic mutations are associated with pleuropulmonary blastomas; these include germ line DICER1 mutation (loss of function) in familial cases, gains of chromosome 8 (most consistent chromosomal abnormality), trisomy 2, unbalanced translocation between chromosomes 1 and X, and p53 mutations or deletions [2].

Clinically, the patient may present with chest or upper abdominal pain, fever, dyspnea, cough, hemoptysis, anorexia, malaise, or neurological symptoms resulting from brains metastasis [1]. Imaging studies have shown varied appearances depending on the tumor type; these include unilocular cysts, a multicystic structure, a cyst containing a polypoid mass, and solid-cystic or entirely solid masses of variable sizes located peripherally in the lung with or without involvement of the pleura or chest wall and may fill the entire hemithorax [1, 5]. The entire thorax should be scrutinized for the presence of any asymptomatic cysts which could be forerunner of type I tumors [5].

Fine needle aspiration cytology of the primary tumors is usually not helpful in reaching a diagnosis. Image guided biopsies when planned should yield multiple needled cores keeping in view the characteristic morphological diversity of the tumor. A solitary needle biopsy core may sample a single tissue type with relatively bland nuclear features or may sample the portion of cyst wall that has discontinuous subepithelial condensation of cells (a diagnostic feature of PPB type I), hence concealing the true nature of the disease, as occurred in this case. Therefore, tumor resection and histological examination should be undertaken as a standard procedure to reach a confident definitive diagnosis on which appropriate aggressive therapy will be based [1, 5].

Three types of the PPB have been described based on morphology. Type I tumors carry the most favorable prognosis and account for 15% to 20% of all PPB, whereas type II and type III tumors behave aggressively and together (distributed equally) account for 80% to 85% of all PPB. These three tumor types form a continuum with progression over time from type I to type III tumor [4].

Type I tumors are purely cystic, peripherally located, show striking absence of chest wall invasion, and may be single or multicystic and the cyst wall is usually thin without grossly visible solid areas [6]. On microscopic examination, the cysts may be round- to oval-compressed and lined by cuboidal or columnar ciliated epithelium; the subepithelium may contain a continuous or discontinuous but characteristic layer of primitive round- to spindle-shaped cells reminiscent of cambium layer of sarcoma botryoides with scattered few rhabdomyoblasts. The primitive cell condensations in the subepithelium may be so scanty and focal that thorough sampling of the cyst wall may be required to locate them; moreover, finding of nodules of immature cartilage should raise suspicion of PPB and merit thorough sampling of the specimen. The differential diagnosis for PPB type I includes congenital pulmonary airway malformation (CPAM) and fetal lung interstitial tumor (FLIT). CPAM is a benign cyst without subepithelial malignant cell condensations or immature cartilage. FLIT is a newly recognized entity with some overlap with type I pleuropulmonary blastoma and is characterized by irregular airspaces enclosed by irregularly thickened septae composed of immature mesenchyme and lined by polygonal cells with underlying thin smooth muscle layer without cambium layer of blastema-like cells [1, 5–7].

Type Ir (type I-regressed) tumors are cystic containing few spindle shaped cells in the cyst wall with few foci of dystrophic calcification but without subepithelial malignant cell condensation. It might represent a regressed or a genetically destined but abortive type I tumor. Only 8% of such tumors show onward progression to PPB type II or type III. Its differential diagnosis is the same as for type I tumors [5].

Type II tumors are partly solid and partly cystic, therefore sharing features of both type I and type III tumors. The cysts may be visible grossly or microscopically; if the cysts are seen only microscopically the tumors are called predominantly solid type II tumors, but if the tumor is largely cystic it is called predominantly cystic type II tumor. Microscopically these cysts are identical to the cysts of type I tumors, whereas the solid portions show microscopic features identical to type III tumors. Predominantly cystic type II tumors are to be differentiated from type I tumors; the aforesaid tumors have plaque-like or nodular proliferation in the cyst wall composed of blastema-like cells or malignant spindle cells with or without rhabdomyoblasts in the subepithelium [4–7].

Type III tumors are entirely solid tumors usually presenting as large, well-circumscribed masses partly filling the hemithorax with or without attachment to the chest wall or mediastinum. The tumor may be friable due to hemorrhages and necrosis. Microscopically, there are blastematous foci in which the constituent cells have scanty cytoplasm, round to ovoid nuclei with granular chromatin, inconspicuous nucleoli, and frequent mitoses; their stroma is less dense and fibroblastic that blends with sarcomatous component. The sarcomatous component is composed of spindle cell proliferation arranged in fascicular pattern; their nuclei show anisonucleosis and hyperchromasia with scattered pleomorphic bizarre giant cells. Such anaplastic bizarre cells are a feature of type III and some type II tumors, but they are not found in type I tumors. There are scattered polygonal or elongated rhabdomyoblasts (some having cytoplasmic cross-striations) occurring singly or in clusters and sheets. Also, there is a variable proportion of immature to mature hyaline cartilage; the cartilage component may be small or large in amount and the chondrocytes may show a degree of cellularity and pleomorphism to the extent of chondrosarcoma. There may be areas of infarction and necrosis affecting the cartilaginous, blastematous, or sarcomatous component producing pseudocysts (not lined by epithelial cells); similarly, there can be foci of myxoid degeneration. Due to the presence of the malignant spindle cell component, the differential diagnosis for type II and type III tumors includes primary or secondary rhabdomyosarcoma, malignant teratoma, synovial sarcoma, other spindle cell/undifferentiated sarcomas, or pulmonary blastoma, whereas due to the presence of primitive blastema, the differential diagnosis includes metastatic Wilm's tumor. Location, morphology, imaging studies, and immunohistochemistry are helpful in making this differentiation [4–7].

Immunohistochemical staining is not essential for diagnosis but is supportive of the morphological diagnosis. Desmin and muscle specific actin are usually positive in the obvious rhabdomyoblasts and also in few small cells of cambium layer (subepithelial condensation). The blastema cells are weakly positive for muscle specific actin and neuron specific enolase but are negative for WT1. The mature and immature chondrocyte nuclei stain for S100. Cytokeratin stains epithelial cells lining the cysts. Vimentin stains rhabdomyoblasts, chondrocytes, and other foci of sarcomatous proliferation. Proliferation markers like Ki67 show high level of positivity in all cell types [1, 4, 5].

The commonest site for metastatic spread is the brain [4]. The metastatic deposits are characterized by simplified histological appearances as compared to the diverse morphology of the primary tumor. These metastases are composed of either rhabdomyosarcoma or undifferentiated spindle cell sarcoma without cysts or cartilaginous component. Therefore, it is mandatory to follow up these patients by brain imaging studies [4]. The long term survival of more than 80% has been reported for type I and less than 50% for type II and III tumors [4]. Type I tumors progress over time to type II and type III tumors [4, 5, 7].

The recommended treatment for type I tumors consists of surgical excision and adjuvant chemotherapy. For type Ir tumors only follow-up but no chemotherapy is recommended. For the usual type II and type III tumors, the treatment consists of aggressive surgery and chemotherapy. For large type II and type III tumors, after initial confirmation by multiple needle core biopsies, 2 to 4 courses of neoadjuvant chemotherapy are instituted reducing the tumor size usually by more than 90%, followed by surgical resection. The recommended chemotherapeutic agents are ifosfamide, vincristine, actinomycin D, and doxorubicin (IVADo regimen). For brain metastasis, the recommended treatment consists of all three modalities, that is, surgery, radiation therapy, and chemotherapy in an attempt to achieve cure [5, 7].

For recurrent tumors high dose consolidation therapy (HDCT) with autologous stem cell rescue (ASCR) is recommended. Studies have shown that there is no additive advantage of radiation therapy following surgery and chemotherapy. Therefore, radiation therapy is reserved for patients with known but nonresectable tumors or for residual tumors after chemotherapy [5].

In summary, the clinicians and pathologists should have a high index of suspicion for lung cysts that present in pediatric age. Also the patients, their siblings, and their first degree relatives should be screened for associated benign and malignant conditions.
